# Mechanisms of entanglement: how a gendered world makes a gendered brain

**DOI:** 10.1186/s13293-026-00872-2

**Published:** 2026-03-08

**Authors:** Gina Rippon

**Affiliations:** Institute of Health and Neurodevelopment, Aston University, Birmingham, B4 7ET UK

**Keywords:** Sex, Gender, Sex/Gender, Social cognitive neuroscience, Social brain, Social rejection

## Abstract

Abstract. Contemporary understanding of key neural processes has advanced the study of the dynamic, iterative influences between the brain and external events, contributing to a growing evidence base concerning the entanglement between human brain structure and function and socio-cultural contextual factors, with consequent behavioural implications. This is particularly relevant to any understanding of differences in apparently sex-linked human behavioural phenotypes and the role of external factors in producing such differences. Relevant insights are provided not only by the relatively well-established concept of experience-based neuroplasticity, but also by research into the brain-changing effects of social context, which can include gendered attitudes and expectations. The developing study of the socially embedded brain offers a powerful organising framework to inform both methodological and theoretical approaches to an understanding of the brain-based mechanisms of biology/society interactions. Additionally, the emerging application of models of predictive coding processes in the brain to human social behaviour potentially offers wide-ranging insights into the role of rule-based, socio-culturally determined, lived experiences in shaping brain development and function and tracking. This paper aims to demonstrate how this framework could be harnessed in neuroscience research into the dynamic entanglement between sex-related brain processes and social contextual influences such as gender.

## Sex/gender entanglement: emergence of a social context model

In a paper in 2014, Rippon et al. proposed key principles to be acknowledged in neuroimaging research into sex and gender [1]. The focus was on research to advance the understanding of the interaction between the neurobiology of individuals and the cultural environment in which they develop and function. The concept of ‘sex’ was taken to refer to the biological attributes — chromosomes, gonads, genitalia —typically categorized as male or female. With respect to the cultural environment, the focus was on ‘gender’, broadly referring to the social, cultural, and psychological roles, behaviours, and expectations that societies constructed around female-male differences.

The aim of the set of recommendations concerning research design, analysis and interpretation was to move beyond experimental designs that focussed on biological sex as a fixed, binary, and deterministic independent variable, and to urge additional attention to gender-related variables, acknowledging the role of social context in both neural and behavioural variation.

One aim was to characterise the shortfall in the traditional “essentialist” models of experimental design, based on an unchallenged notion of sex differences in brain and behaviour being biologically determined, inevitable and invariant. At the time of writing, the emergence of the concept of neuroplasticity was noted as offering evidence that many such differences may not be pre-determined and inevitable but may reflect the brain’s lifelong adaptation to external social, cultural, and environmental influences, thus blurring the line between what is “biological” and what is “socially constructed.” There was, for example, early evidence of the effects on adult human brains of skill learning and environmental events (see early demonstrations in adults such as taxi-driving or juggling) [2]. The notion that such flexibility was lifelong was relatively new; previous models of brain development has incorporated the notion of its end-product as ‘hard-wired’ [3, 4].

In addition there was evidence of the alteration of brain responses in the context of external, social expectations, dubbed elsewhere as brain ‘permeability’ [5]. When task performance was framed in a negative social context, by referencing stereotypically poor maths performance or spatial ability, for example, increased activation in networks associated with emotional self-regulation or the processing of social feedback occurred, as opposed to the recruitment of cognitively appropriate networks shown when the task framing was positive or neutral [6, 7].

It was clear there was a need to acknowledge that brain variables may not be purely biological, but may incorporate external factors, perhaps those instantiated in an understanding of gender.

**Fig. 1:**
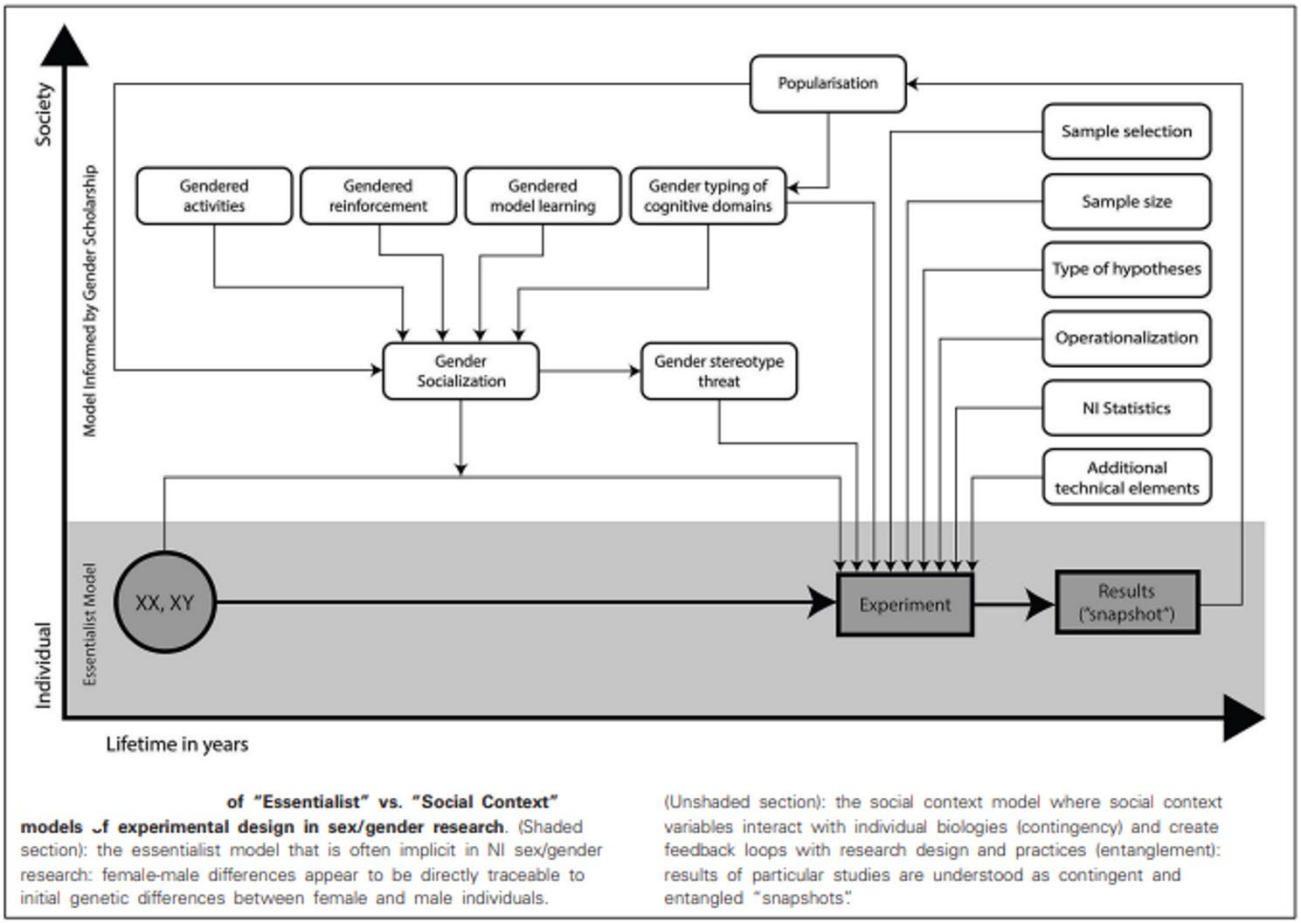
Comparison of "Essentialist" vs. "Social Context" models of experimental design in sex/gender research

A “social context” model was outlined as an alternative to the essentialist approach, drawing attention to external socio-cultural forces that could, iteratively, be affecting female-male differences in both brain and behaviour (See Fig. 1).

The external social forces associated with gender, here very broadly defined to include gendered socialisation, encompassing, for example, gendered reinforcement and gendered activities, were nominated as key factors to be accounted for as additional brain-changing forces, inevitably entangled with sex-linked brain development and function. This was linked to the principles identified in the paper as “contingency”, the gendered social contexts within which behaviour occurs and “entanglement”, the emerging evidence that allegedly sex-linked behavioural differences and the underlying neural circuitry will be entangled with the influences from such social context, s. The term ‘sex/gender’ was adopted to reflect the nature of such entanglement [8–10]. 

These were relatively early days for neuroimaging approaches to differences in sex-linked human behavioural phenotypes. A traditional, ‘hunt the difference/blame the brain’, agenda had accompanied the emergence of more widely available brain imaging techniques and their application to such differences, with a continued emphasis of sex-based brain differences as core explanatory models. This was frequently linked to evolutionary arguments, claiming continued recurrence of psychological and behavioural sex differences as due to fixed, biologically based adaptative specialisations associated with the demands of sexual selection and parental investment [11, 12].The notion that ‘context matters’ did not figure strongly in interpretations of those differences that were reported [13, 14].

Wide-ranging critiques of such essentialist models revealed major shortfalls in attempts to employ them in explanations of observed differences between males and females, where there was clear evidence of marked within-group heterogeneity and pronounced overlap between them [15]. Similarly, major methodological weaknesses in studies supporting claims for innate, hard-wired differences were identified, including small sample sizes, reverse inferences and false positives. Additionally, there was limited acknowledgment of the role of neuroplasticity, or of issues associated with social identity [16–18]. These criticisms were more recently supported by a significant review of several decades of sex/gender brain imaging studies which failed to find any consistent or meaningful female-male differences in the brain [19].

Alternative, social constructionist, explanations suggested that biology was broadly irrelevant and that disparities between males and females were the result of cultural norms, social expectations and patriarchal structures. This has been described as a ‘blank slate’ approach, assuming minimal or no innate differences, with disparities arising through variations in post-natal developmental histories, via the primacy and power of cultural and social forces [20, 21]. The two approaches were generally considered mutually exclusive, with context irrelevant in essentialist arguments, as opposed to constructionist claims of the deterministic power of social and cultural forces.

Both philosophical and empirical challenges emerged to these approaches. For example, Fine et al. (2017) argued that the apparent stability of sex differences over time could well be due to constraints imposed on human biological flexibility by extant cultural stereotypes and expectations [22]. Sex-differentiated environmental processes (such as can be found in many examples of gendered socialisation) may interact with a generic, inherited, ability to perceive and respond to such processes, possibly from a very early stage of development. The resultant sex-differentiated phenotype is thus the product of cultural forces rather than genetic programming. The key point of this article was that adaptations could be *culturally* inherited, as well as genetically inherited, and that the environment could be a source of stability and biological sex a source of variation, rather than the other way around. This could result in the extent of within group variability and cross-cultural variation that purely essentialist arguments had failed to address.

Key areas of human brain research have similarly put forward experimental models that have shifted the focus from biological sex as primary causal drivers in group differences, to the power of social context in determining such differences. Social endocrinology, for example, asserts that hormones and social context are mutually influential. Van Anders (2013) provides evidence that sex differences in hormones and behaviour are inextricably linked with social identities and norms [23]. In Testosterone Rex, Fine (2017] has challenged the idea of testosterone as a primary driver of sex/gender differences in behaviour. Citing evidence from neuroscience, psychology and evolutionary biology, she demonstrates that human behaviour is far more flexible and influenced by context, learning and culture than biological determinism suggests [24]. In affective neuroscience, Barrett (2017) argues that emotions are not produced by distinct, hard-wired biological circuits but are ‘constructed’ by the brain, using past experience, context and cultural concepts [25]. While not directly addressing the issue of sex differences, this theory of constructed emotion is of relevance to counteracting essentialist arguments about innate neurobiological dichotomies in emotional behaviours such as empathy and nurturance [3].

To date, within sex/gender cognitive neuroscience, the emergence of models of the social brain has served as the main forum for exploring mechanisms underpinning the entanglement of brain processes with external social forces such as gendered norms and expectations. This takes forward the generic acknowledgment of experience-based neuroplasticity or the brain-altering effects of social context, as outlined in the Rippon et al. paper [1], and furthers the understanding that brain development and organisation are lifelong and socially embedded processes, such that gendered meanings and opportunities plausibly shape both behaviour and the neural circuitry that supports it.

## The socially embedded brain

A core premise of the social brain theory is that the human brain has evolved to enable and ensure social interaction [26]. Human brains are equipped with networks to support the processing of the behaviour of others, and the understanding of the rules and norms that underpin successful social interaction. Higher level social processing underpins the acquisition, encoding and retention of social knowledge and, in interaction with key motivational processes, enables the production and regulation of socially appropriate behaviour. Studies based on these premises have enabled the identification of key cortical structures and networks and their links to various aspects of social behaviour (see Table 1).

**Table 1:** Cortical correlates of core functions of the social brain with links to social behaviour

Structure/Network.	Core functions	Links to social behaviour
Medial Prefrontal Cortex (mPFC) [27]	Self-referential processing; impression formation	Inferring others’ traits and intentions; evaluating social norms; social decision-making [refs]
Temporo-Parietal Junction (TPJ) [28, 29]	Theory of Mind (mind reading); mental state attribution	Predicting behaviour in social contexts
Posterior Superior Temporal Sulcus (pSTS) [30, 31]	Biological motion; intentional action coding	Interpreting social signals; recognising communicative intent
Posterior Cingulate/Precuneus [32, 33]	Self-other reflection; autobiographical memory	Social identity; perspective taking; mentalising about self and others.
Amygdala [34]	Salience detection; threat and reward evaluation	Rapid evaluation of fear/anger in others; emotional facial expressions.
Ventral striatum (inc. nucleus accumbens) [35]	Reward and reinforcement learning	Experiencing pleasure from co-operation, approval, affiliation
Orbitofrontal/ventromedial PFC (OFC/vmPFC) [36]	Value representation; context-specific updating	Social reward/punishment learning; fairness evaluations
Dorsal Anterior Cingulate Cortex (dACC) [37, 38]	Conflict monitoring, error evaluation	Experience of “social pain” during rejection/exclusion; regulation of Go/NoGo response to social feedback
Anterior insula [39]	Awareness of bodily state	Visceral awareness during social threat; empathy for others’ pain
Dorsolateral PFC (dlPFC) [40]	Executive control; behavioural regulation	Application of social rules; appraisal of rejection
Default Mode Network (DMN: mPFC, TPJ, PCC) [41]	Mentalising; self-refererence	Predicting social outcomes; scripting interactions
Salience network (dACC, Insula) [40]	Detecting relevance/social cues; switching attention	Highlighting socially significant events (e.g. rejection, approval)
Executive control network (dlPFC; parietal) [42, 43]	Goal-directed control; application of social rules	Regulating responses to conflict; co-ordinating co-operation

In relation to sex/gender entanglement, these findings offer an opportunity to examine how allegedly sex-linked patterns of brain function can interact dynamically with gendered social forces, and to specify the mechanisms through which these interactions shape behaviour. Progress in this area depends on identifying a neurobehavioural social process that can serve as a proxy for the broader interplay of sex and gender.

## Belongingness and Social Rejection as a window into sex/gender entanglement

In a recent paper, Rippon (2023) identified the theory of ‘belongingness’ as offering a core construct within social cognitive neuroscience, providing a bridge between neural processes and social experience [44]. This was in the context of outlining a more contemporary, interdisciplinary, approach to research into persistent gender gaps in many spheres of performance and achievement.

Belongingness refers to the fundamental human need to form and maintain enduring, positive, and significant, social relationships [45]. Its origin is as a motivational theory of social behaviour, and it is claimed to apply across cultures, ages and contexts. Social cognitive neuroscience has operationalised the theory using studies of social exclusion, showing that the experience of belonging (or not) is instantiated in specific brain networks. There is additional evidence of sex/gender differences in rejection sensitivity, at both the brain and behaviour level [46, 47]. As there is considerable evidence in social and organisational psychology of extensive gendered experience of social exclusion or ostracism (44), it is proposed that this framework could provide a useful lens to apply to the issue of sex/gender entanglement.

Stereotypical attitudes can function as a set of social scripts that regulate who is (or is not) granted belonging in particular contexts and under what conditions—for example, whether women are perceived as fitting in technical domains or whether men are accepted in nurturing roles. Environmental cues signalling potential exclusion can result in avoidant behaviour [46]; experimentally manipulated stigmatisation can negatively affect motivation and achievement; [47]; the experience of social rejection is associated with low levels of self-esteem [48], with additional evidence of sex/gender differences in this association [48–50].

In social cognitive neuroscience, the role of belonging as a social motivational force has been operationalised via scanning tasks inducing experiences of social rejection. These include Cyberball, an online ball-tossing game where participants are intermittently excluded [51], or a contrived scenario involving ‘accidental’ exposure to negative personal assessments [52, 53]. These studies of social rejection have demonstrated that inclusion activates reward-related regions such as the ventral striatum, while exclusion or rejection recruits the dorsal anterior cingulate cortex and anterior insula, core parts of the salience network determining responses to socially significant events [54–57](see Table 1).

It has been suggested that there is a ‘social pain’ network equivalent to that activated by physical pain, reflecting some kind of neural ‘sociometer’ monitoring positive and negative social experiences [50, 58], although this equivalence has recently been challenged [59]. But it is clear that social rejection engages those systems in the social brain which underpin emotional regulation, negative affective coding, and behavioural inhibition (see Table 1).

It is also clear that this is an iterative process, both cortically and behaviourally. Repeated rejection has been shown to alter neural sensitivity over time — especially in adolescence, when social evaluation strongly shapes brain development [60]. A history of peer victimisation has been shown to be associated with altered amygdala -vlPfC connectivity in social rejection tasks [61, 62]. A recent world-wide study reported that long-term exposure to the manifestations of gender inequality was associated with significant sex-differences in key brain structures [63]. Contemporary studies of the brain bases of social behaviour have thus advanced the understanding of the role of neural plasticity, as identified in Rippon at al (2014), here linked to the brain-changing effects of social factors in the interaction between sex and gender.

Associated with these cortical changes, social rejection can elicit a wide spectrum of behavioural responses, including aggression and lowered hostility thresholds [64], withdrawal and disengagement [65] and increases in rejection sensitivity, “a tendency to anxiously expect, readily perceive and intensely react to cues of interpersonal rejection” [47, 66]. Social rejection can also elicit regulatory strategies such as self-silencing, in which individuals suppress their own needs or voices in order to maintain valued relationships [67]. Research indicates that this pattern of behaviour is more common in women, therefore relevant to understanding increasing gender gaps in areas of power and achievement [68].

As with alterations to neural sensitivity described above, the behavioural effects of social rejection show the same iterative effects, with the variations in self-esteem and rejection sensitivity associated with social rejection shown to modulate cortical responses to social feedback, with consequent feedforward effects on behavioural responses. As above, sex/gender differences in this process have also been reported, with stronger patterns of activation in females [68–70].

As well as the negative effects of a *lack* of social interaction, additional research into social motivational processes has demonstrated the positively rewarding aspects of such interactions. This has been characterised, for example, via the use of joint attention paradigms, operationalising engagement in shared social realities. Achievement of successful shared attention was strongly associated with increases in neural activation in reward-related brain areas [71]. There is evidence of greater striatal involvement in women when the joint attention paradigm involves social reward [72].

Rippon et al. (2014) stressed the role of neural plasticity in the dynamic interaction between sex and gender. Contemporary studies of the brain-changing consequences of social experiences provide a powerful evidence base for this process. Additionally, more recent developments in neuroscience offer the intriguing possibility of identifying detailed neural signatures specific to such social experiences.

## Predictive coding and the social world

Predictive coding has emerged as a unifying framework for understanding how the brain processes information, integrating perception, action, and cognition under a common principle of prediction and error correction. At its core, predictive coding models propose that the brain is not a passive receiver of sensory input but an active inference system: higher-order cortical regions generate predictions about incoming signals, while lower-order regions register mismatches (prediction errors) that are used to update internal models [73, 74]. This hierarchical exchange of top–down predictions and bottom–up errors allows the brain to minimise surprise and optimise interaction with its environment.

Applied to social behaviour, predictive coding suggests that individuals do not simply react to others’ actions and expressions but anticipate them on the basis of prior experience, cultural scripts, and social learning [75]. In this view, phenomena such as stereotyping or norm enforcement can be understood as the brain’s attempt to minimise uncertainty in the social world by relying on probabilistic expectations about how people will behave. Crucially, predictive models are shaped by cultural and historical forces: gender norms, for example, provide powerful priors that structure expectations about who belongs in particular roles, how others are likely to act, and which responses are socially sanctioned. When reality violates these expectations—such as when individuals transgress gender norms—prediction errors can trigger neural and behavioural responses as outlined above, ranging from heightened attention and affective arousal to exclusion or withdrawal [76, 77].

Thus, predictive coding offers a framework for linking neural computation with the cultural organisation of gender, highlighting how brains and societies co-construct social realities.

Social neuroscience research is identifying the neural correlates of the predictive coding process in different aspects of social behaviour, including violation or conformation of social expectations or the positive or negative consequences of social decision-making [78]. The reward pathways identified in the social brain, especially the striatum and the insula, form part of a core prediction error processing circuit, as well as the anterior cingulate. This could serve as a ‘neural hub’ where predictions concerning social activities could be matched against the consequences, and current and future behaviour regulated accordingly [79]. Using detailed ‘signatures’ of brain activity based on different brain frequencies, it has proved possible to track the emergence in the brain of such predictions and their refinement through experience with external factors, as well as alterations in the face of prediction errors, when input does not match expectations [80, 81].

The early focus of predictive coding empirical research has mainly been on sensory processing, although Kessler and colleagues (2016) outlined its potential application to the study of the atypical social behaviour characteristic of autism spectrum disorder [82]. There is now a small but growing literature that offers the possibility of framing gender stereotypes as priors in predictive coding terms. Brown and Brüne (2012) argue that the brain’s forward modelling and prediction error mechanisms, well established in sensory and motor domains, also apply to social cognition, such that individuals continuously generate social predictions about others’ intentions, actions, and emotions [83]. These predictions, when violated, give rise to distinct “social prediction errors” that drive updating of mental models. Thus stereotypes, in the shape of expectations or predictions, may actually inform the generation of priors in social behaviour [84, 85].

Kelly et al. (2019) extend this framework by situating predictive processing within a sociological account of the self and society [86]. They argue that predictive models of social life are scaffolded by cultural stereotypes and social practices, which act as priors, shaping how the brain anticipates and interprets social events. From this perspective, gendered norms and stereotypes can be understood as culturally transmitted priors: they structure expectations about competence, relationality, or authority, for example, and their violation produces heightened prediction errors. Villiger (2023) takes this one step further and proposes that predictions or expectations could serve as self-fulfilling prophecies, where the perceptions and actions driven by the predictive prior could serve to sustain the pre-existing beliefs and reinforce existing stereotypes [85]. It should be noted that such approaches could be linked to the arguments put forward by Fine et al. (2017), that sex-linked behavioural adaptations can be culturally inherited [22]. This could be characterised as powerful priors determining behaviour- and brain-shaping predictions and consequent responses.

There is evidence of how the beliefs that determine predictions might be acquired. Spiers et al. (2017), using fMRI, employed a prejudice learning task to demonstrate the acquisition of the valence of different hypothetical social groups, primed to be perceived as negative (“stole a drink from a shop”) or positive (”gave their mother a bouquet of flowers”) [87]. They demonstrated that activity in the left anterior temporal cortex (ATL)- a hub for semantic and social knowledge - reflects the accumulation of biased associations during the formation of prejudiced beliefs. fMRI evidence showed that ATL activity increased as participants acquired prejudiced attitudes, suggesting that prejudice emerges from the same integrative processes underpinning semantic memory and social evaluation. The acquisition of negative information was associated with activity in the ventral striatum and the anterior cingulate cortex/dorsomedial prefrontal cortex, core structures underpinning social reinforcement, regulation of social behaviour and the application of social rules (see Table 1), as well as members of the predictive coding circuits identified above [78]. When the expectations generated by evolving stereotypes were violated (e.g., a member of the negative group giving his mother flowers), heightened levels of activity were shown in the associated networks. Within a predictive coding framework, this finding can be interpreted as evidence of how the brain encodes and updates social priors about groups: once established, these priors bias prediction of others’ behaviours and generate prediction errors when individuals act counter to stereotype, thereby reinforcing or modifying prejudiced expectations.

Currently there is no good evidence for sex/gender differences in the core predictive-coding architecture of the brain per se. However, sex-linked differences in anticipatory anxiety, for example, or greater sensitivity to social rejection, could well be framed in terms of predictions differentially weighted by experience-dependent priors or context-sensitive developmental experiences, determined by, for example, gendered socialisation [47]. Measures of these variables could be incorporated into future experimental designs.

Predictive coding offers a metric that can model the dynamic nature of sex/gender entanglement. It could well capture the entangled processes revealed by the study of the social brain and the effects of gendered social experiences and expectations, as described throughout this paper. A key aspect of this approach with respect to the proposed model of entanglement is the consistent evidence of the significant role of cultural context and/or social experience in eliciting any evidence of sex/gender differences.

## Limitations

It must be acknowledged that much of both the theory and the empirical evidence informing this paper remains couched in mainly binary assumptions of both sex and gender. This reflects the state of the evidence base to date. It could well be possible to reconstruct parts of the argument here to acknowledge, in particular, the effect of *individual* differences in cortical and hormonal profiles, and how these interact with a social world. The limited nature of the existing database constrained explanations and exemplars in this paper, but the emerging principles should certainly be applicable to non-binary populations, in both the cortical and the behavioural sense of the word, and, indeed, viewed through a non-binary lens where possible. An acknowledgment of the need for a non-binary approach to reflect social realities, as well as the enrichment offered by such an approach, should be part of any ongoing developments in this sphere (see Next Steps).

In particular, it should be possible to harness the concept of the mosaic brain, that the brain is a patchwork of features, not uniformly male or female, but a unique mosaic of characteristics influenced by both sex-linked biology and social experience, a clear endpoint of the processes discussed above [88]. Indeed, the mechanisms of entanglement proposed here could track the emergence of the mosaic brain itself.

It must also be acknowledged that gender has been broadly characterised to date as a rather generic social construct, framed in terms of cultural meanings and expectations. This overlooks the enormous recent strides in examining both the definition and the meaning of gender. Eliot and colleagues, for example, identify key measurable gender variables that can be incorporated in neuroscience research (such as income, caregiver stress and bias) and, importantly, stress the importance of measures of gendered lived experience [89, 90]. There has been tremendous progress in the development of new instruments to measure gender-related variables [91–93] which will bring a welcome level of nuance to future studies, widening the evidence base for the model proposed here.

## Next steps

Given that studies of the social brain have successfully operationalised many stages of social behaviour, harnessing the insights provided by predictive coding studies could well offer direct tests of the mechanisms of entanglement proposed here. Accepting gender stereotypes as extant social priors, a social perception task could be designed that involved the anticipation of, for example, the membership of more or less stereotypically gendered occupations, with exemplars manipulated to challenge or violate such priors, thus generating a social prediction error. Or, scanner-based tasks could serve as a proxy for gendered experiences [46]. Neural correlates of consequent prediction errors could be tracked using time-sensitive imaging techniques, focussing on core social brain areas, such as the ACC, the insula and the striatum. Behavioural correlates could be explored by confidence ratings, or self-reported level of discomfort/surprise in the face of task outcomes. Ideally, participants in such a study would incorporate a broad range of gender identities as well as relevant lived experience. Additionally, measures of gender-related social variables, such as rejection sensitivity, social anxiety or self-esteem, as well as factors such as income, or caregiver stress would provide crucial (and valid) measures of contextual influences [89].

As outlined above, non-binary approaches to both sex-based and gender-linked variables will greatly extend the applicability of the sex/gender entanglement model proposed here. The resultant data complexity can be addressed by the emergent use of AI models, for example embedding neural time series with behavioural trajectories and social scenarios to demonstrate the interplay between the brain and its world, as well as matching these up with individual differences in personal, social and experiential characteristics. Computational models are explicitly designed to incorporate continuous cortical, behavioural, experiential and social variables, increasingly available via contemporary improvements in assessment tools and to model changes in brain activation as a function of such variables [94, 95]. However, it is necessary to be aware of the dangers of the continued use of binary ‘sex-stratification’ within machine learning algorithms, potentially reinforcing unidirectional biological sex essentialism, and thus undermining efforts to explore the multivariate contributions to mechanisms of sex/gender entanglement. It is crucial that the perceived or allegedly evidenced sex/gender differences that drove traditional analytical approaches are avoided in AI-informed advances [96].

## Summary and Conclusion: 3 Ps – how the world leaves its imprint on the brain

The model proposed here rests on three principles of brain function, that of plasticity, the evidence of a lifelong ability to flexibly adapt to external events; of permeability, demonstrating the iterative impact of social influences on all forms of human brain processes; and, finally, that of prediction, with the brain acting as an active inference system, generating rule-based predictions based on prior experience of external events [5].

Taken together, these insights point toward a new research programme in which gendered cultural scripts could be identified as active priors in the formation of a brain’s predictive models of the social world. This perspective moves beyond static debates about “biology versus society” to show how lived experience and cultural expectations literally shape neural circuitry across the lifespan. It also offers testable hypotheses: when gendered priors are violated the brain’s prediction-error systems may register surprise or threat, accompanied by associated psychological consequences on, for example, sense of belonging, or self-esteem.

Future research can capitalise on this framework by combining time-sensitive neuroimaging, behavioural markers of prediction error, and more nuanced measures of gender as a sociocultural variable, based on continuous rather than categorical variables. By doing so, neuroscience can begin to map how social forces leave their imprint on the brain, and, crucially, how these imprints reinforce or erode persistent gender gaps in education, work, and wellbeing. The entanglement model therefore not only reshapes the science of sex/gender but also points to concrete implications for equity, inclusion, and human potential [97, 98].

## Data Availability

No datasets were generated or analysed during the current study.
